# PE/PPE Proteome and ESX-5 Substrate Spectrum in *Mycobacterium marinum*

**DOI:** 10.3390/ijms25179550

**Published:** 2024-09-03

**Authors:** Lili Yan, Hiu Ying Lai, Thomas Chun Ning Leung, Hiu Fu Cheng, Xin Chen, Stephen Kwok Wing Tsui, Sai Ming Ngai, Shannon Wing Ngor Au

**Affiliations:** 1 School of Life Sciences, The Chinese University of Hong Kong, Shatin, Hong Kong 999077, China; 2 School of Biomedical Sciences, The Chinese University of Hong Kong, Shatin, Hong Kong 999077, China

**Keywords:** ESX system, proteomics, PE/PPE protein families

## Abstract

PE/PPE proteins secreted by the ESX-5 type VII secretion system constitute a major protein repertoire in pathogenic mycobacteria and are essential for bacterial survival, pathogenicity, and host–pathogen interaction; however, little is known about their expression and secretion. The scarcity of arginine and lysine residues in PE/PPE protein sequences and the high homology of their N-terminal domains limit protein identification using classical trypsin-based proteomic methods. This study used endoproteinase AspN and trypsin to characterize the proteome of *Mycobacterium marinum.* Twenty-seven PE/PPE proteins were uniquely identified in AspN digests, especially PE_PGRS proteins. These treatments allowed the identification of approximately 50% of the PE/PPE pool encoded in the genome. Moreover, EspG5 pulldown assays retrieved 44 ESX-5-associated PPE proteins, covering 85% of the PPE pool in the identified proteome. The identification of PE/PE_PGRS proteins in the EspG5 interactome suggested the presence of PE–PPE pairs. The correlation analysis between protein abundance and phylogenetic relationships found potential PE/PPE pairs, indicating the presence of multiple PE/PE_PGRS partners in one PPE. We validated that EspG5 interacted with PPE31 and PPE32 and mapped critical residues for complex formation. The modified proteomic platform increases the coverage of PE/PPE proteins and elucidates the expression and localization of these proteins.

## 1. Introduction

*Mycobacterium tuberculosis* (*Mtb*) is the etiological agent of tuberculosis, which causes 1.5 million deaths annually worldwide [1]. *Mtb* has a complicated type VII secretion system, classified as ESX-1 to ESX-5, and they are responsible for the export of effectors across the cell membrane. ESX-1, -3, and -5 are critical for pathogenicity and host–pathogen interactions [2,3,4,5]. ESX-1 is the best-characterized secretion system and is implicated in host phagosomal permeabilization and cell apoptosis [6,7]. ESX-3 is involved in metal homeostasis, phagosome maturation, and host innate immunity [3,8]. In slow-growing mycobacteria, PE and PPE proteins are encoded by 8–10% of the genome. Most PE/PPE family members are secreted by the ESX-5 type VII secretion system, which contributes to cell viability, nutrient uptake, and immune responses [9,10].

PE and PPE proteins contain proline-glutamic acid and proline-proline-glutamic acid motifs in the N-terminal domains. Although the N-terminus is highly conserved, the length and amino acid sequences of the C-terminal domains vary widely. *Mtb* has 35 PE proteins, 65 PE-PGRS (a common subset of PE proteins with polymorphic GC-rich sequences in the C-terminus), and 70 PPE proteins [11,12]. Although the corresponding genes are scattered across the genome, 28 are clustered in operons [13], including *pe*35/*ppe*68, *pe*36/*ppe*69, and *pe*5/*ppe4*, located in the esx-1, esx-2, and esx-3 locus, respectively [13,14,15,16]. The esx-5 locus is duplicated thrice and contains multiple *pe/pp*e genes [2], highlighting the importance of this secretory system for PE/PPE protein export. The analysis of the operon structure of *pe/ppe* genes and coexpression data indicate that PE and PPE proteins function in pairs. To interact directly with host cells, these proteins are either surface-associated or secreted in soluble form. PE/PPE export by ESX secretion systems is coordinated by the chaperone EspG, which binds and stabilizes PPE substrates targeted for secretion [2,17,18,19,20,21]. Up-to-date, three PE-PPE-EspG heterotrimeric structures have been resolved: PE25-PPE41-EspG5, which increases humoral and cellular immune responses [22]; PE5-PPE4-EspG3, involved in iron uptake [3]; and PE8-PPE15-EspG5, implicated in triacylglycerol accumulation under dormancy-inducing conditions [23].

Transcriptomic and proteomic studies have demonstrated that PE/PPE proteins strongly contribute to bacterial pathogenicity [24,25,26,27]. The transcription of *pe/ppe* genes in mycobacteria is regulated by sigma factors, which are associated with host adaptation, cell wall stress response, nutritional stress, and lung hypoxia [28,29,30,31]. In a guinea pig model of *Mtb* infection, PE/PPE proteins identified in infected lung tissues by proteomic analysis comprised the third most abundant functional category [32]. However, the number of secreted PE/PPE proteins found in previous proteomic studies only accounts for 20% of *pe/ppe* genes encoded in the respective genomes, limiting our understanding of their expression and localization [4,26]. These challenges arise from several reasons. First, these proteins have unique characteristics, including high sequence similarity (e.g., PE_PGRS33 and PE_PGRS4 share 70% sequence identities; PPE18 and PPE60 share 80% sequence identities) [33,34]. Second, razor peptides may match with one PE or PPE protein during database searches. Third but perhaps also the most critical issue, most of these proteins contain a few trypsin cleavage sites. Therefore, mapping PE/PPE proteins by classical proteomic approaches using trypsin digestion is challenging [12].

This study characterized the expression and secretion profiles of PE/PPE proteins in *M. marinum* using classical and modified proteomic approaches. *M. marinum* is a pathogenic bacterial species that infects fish and humans. This species is widely used as a surrogate for *Mtb* because of the similarity in genome composition, host–pathogen interactions, and pathogenesis [19,20,35,36,37]. Its genome also has many *pe/ppe* orthologues, including 27 PE proteins, 148 PE-PGRS proteins, and 106 PPE proteins [19]. Here, we compared the PE/PPE protein profiles from trypsin digestion and AspN digestion. We found that, because of the high aspartic and glutamic acid content in PE/PPE proteins, AspN digestion can increase these proteins’ coverage, especially PE_PGRS proteins. In addition, the substrate spectrum of ESX-5 was mapped using the EspG5 pulldown assay. The direct biophysical interactions of the two newly identified EspG5/PPE31 and EspG5/PPE32 pairs were validated by coexpression and pulldown assays. The analysis of the correlation between protein abundance and phylogenetic relationships allowed the creation of an interaction network of PE or PE_PGRS and PPE.

## 2. Results

### 2.1. Proteinase AspN Treatment Improved the Coverage of PE/PPE Proteins

**Figure 1:**
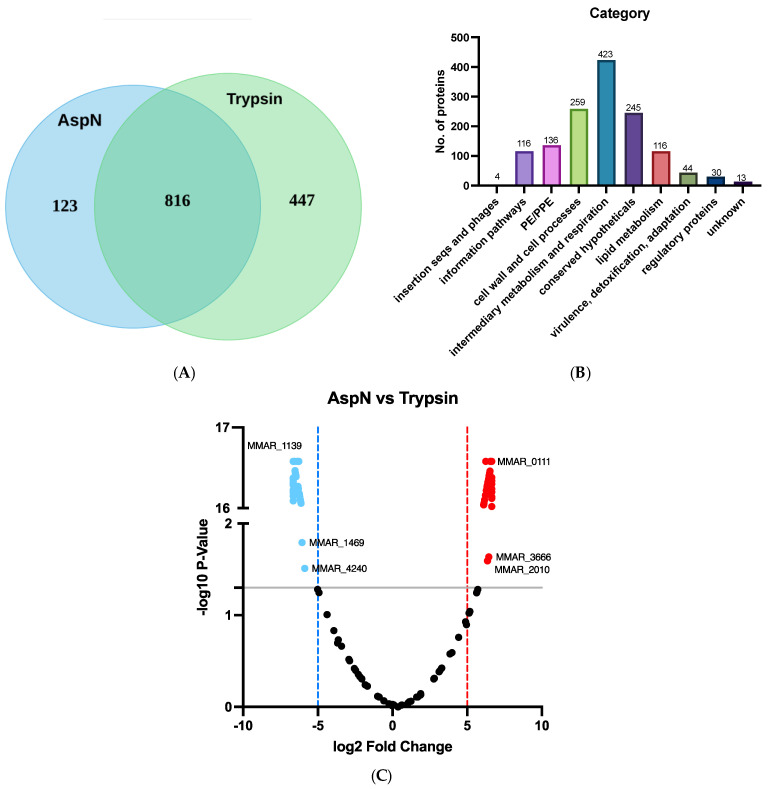
Distribution of the total proteins of the *M. marinum* proteome. (**A**) Venn diagram showing the identified proteins using AspN and trypsin treatment. (**B**) Category of total proteins according to the Mycobrowser (https://mycobrowser.epfl.ch). (**C**) Volcano plot of PE/PPE proteins identified by AspN cleavage and trypsin cleavage. The value of the *y*-axis demonstrates the —log10 *p*-value of the comparison of the performance between AspN and trypsin. The value of the *x*-axis showed the log2 fold change between the trypsin and AspN identifications. The PE/PPE proteins that were significantly detected (*p*-value < 0.05) in the AspN and trypsin digested samples are indicated by red and blue dots over the grey line, respectively.

PE/PPE proteins are critical for mycobacterial cell survival, and their expression and secretion have been shown to be up-/down-regulated during different growth conditions [28]. In this study, *M. marinum* was cultured under laboratory culture conditions until the late-log phase. This allowed us to compare the findings from previous proteomic studies under the same growth phase [4,26]. The proteome of the culture filtrate obtained from trypsin treatment and AspN treatment were compared. Each digested profile was generated from at least three biological replicates. AspN and trypsin treatment yielded 123 and 447 unique proteins, respectively, and 816 were found in both groups (Figure 1A). A total of 1386 proteins from eight functional categories were identified (Figure 1B; Table S1). Most of them are involved in the intermediary metabolism and respiration (30.5%), cell wall and cell processes (18.7%) and conserved hypothesis proteins (17.7%). PE/PPE proteins accounted for 10% of the total. These results indicated that the proteome obtained contained some old lysed bacterial cells and secreted proteins. Overall, the two protein profiles share high similarities in the distribution of isoelectric points and molecular masses (Figure S1; Table S2). It is worth mentioning that 72% had a pI below 6. The acidic nature of the *M. marinum* proteome may be attributed to the presence of PE/PPE proteins since 97% of them had a pI value below 6. The three most acidic proteins we identified were MMAR_2591 (PPE8; pI 3.65), MMAR_2067 (PE_PGRS; pI 3.68), and MMAR_5321 (PE_PGRS; pI 3.75). The smallest and largest PE/PPE proteins were MMAR_0544 (PE5; 9.6 kDa) and MMAR_1639 (PPE; 270.5 kDa), respectively (Table S3).

The two treatments allowed mapping of 13 PE, 71 PE_PGRS, and 52 PPE proteins, accounting for approximately 50% of PE/PPE proteins encoded in the *M. marinum* genome. Among them, 3 PE, 18 PE_PGRS, and 6 PPE proteins were uniquely detected in the AspN profile, indicating that the use of AspN increased the PE/PPE identification by 20% (27/136) (Table 1). We also compared the efficiency of the two proteases in cleaving PE/PPE proteins by volcano plot (Figure 1C; Table S4). Although coverage was similar between the treatments, at *p* < 0.05, trypsin favored toward 2 PE, 15 PE_PGRS, and 15 PPE proteins (blue dots), whereas AspN was found more on 4 PE, 17 PE_PGRS, and 7 PPE proteins (red dots). The differences in their performance were likely related to the number of the cleavage sites available. For example, PE MMAR_0111, having 5-fold higher abundance in AspN identification, has one trypsin cleavage site but seven AspN cleavage sites.

### 2.2. Quantification of PE/PPE Protein Abundance

**Figure 2:**
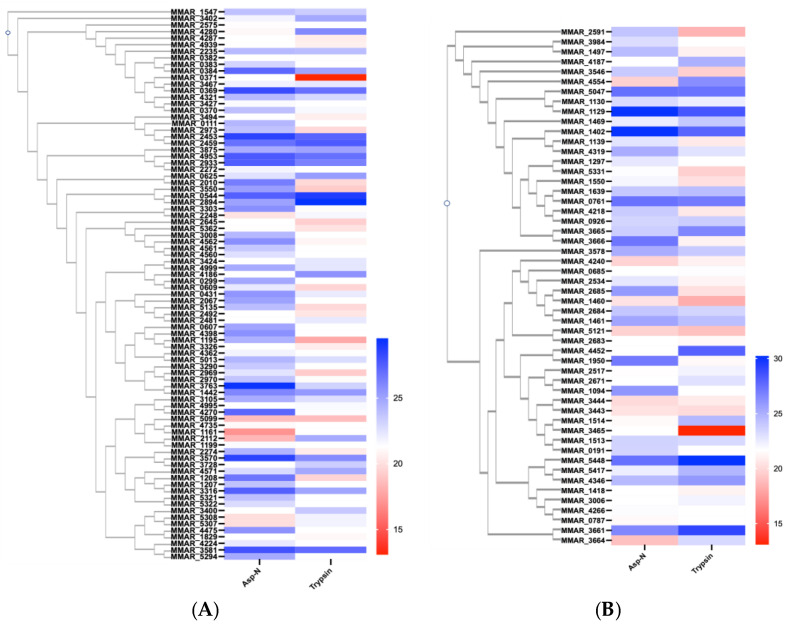
Comparison of (**A**) PE and (**B**) PPE protein abundance from AspN and trypsin identifications were plotted using heatmap. Analysis was scaled by Log2(Abundance) of their source proteins as detected in proteomic analysis. The abundance of PE/PPE proteins is presented using color spectrometry form red (low) to blue (high). The phylogenetic analysis was performed using CLUSTALW. Protein abundances were measured across at least three biological replicates and technical triplicates.

We used label-free quantification to evaluate the expression and secretion of PE/PPE proteins in the *M. marinum* proteome. Protein abundance was calculated by summing all unique peptides of each protein from each treatment and their differences were visualized using heatmaps (Figure 2). In general, the abundance pattern is in line with previous transcriptomic studies [25,38]. This was indicated by the high abundant MMAR_2894 and MMAR_5448 and the low abundant MMAR_0371 and MMAR_1161. We also found that 13 PEs and 14 PPEs shared similar abundance (with fold change < 1) in both protease treatment, in particularly, for proteins encoded by neighboring genes, such as PE_PGRS20 and PE_PGRS2 (MMAR_2969 and 2970) (Figure 2A), and PPE61 and PPE61_1 (MMAR_3444 and 3443) (Figure 2B). The association of gene neighborhood and protein abundance was also shown within one profile, for example with PPE31 and PPE32 (MMAR_2683 and MMAR_2684) in trypsin-treated samples and with PE_PGRS genes (MMAR_4560 and 4561) in AspN-treated samples. Variations of the abundance between the two profiles are consistent with the protease performance toward different PE/PPE as indicated in the volcano plot analysis (Figure 1C).

### 2.3. EspG5-PE/PPE Interactome

**Figure 3:**
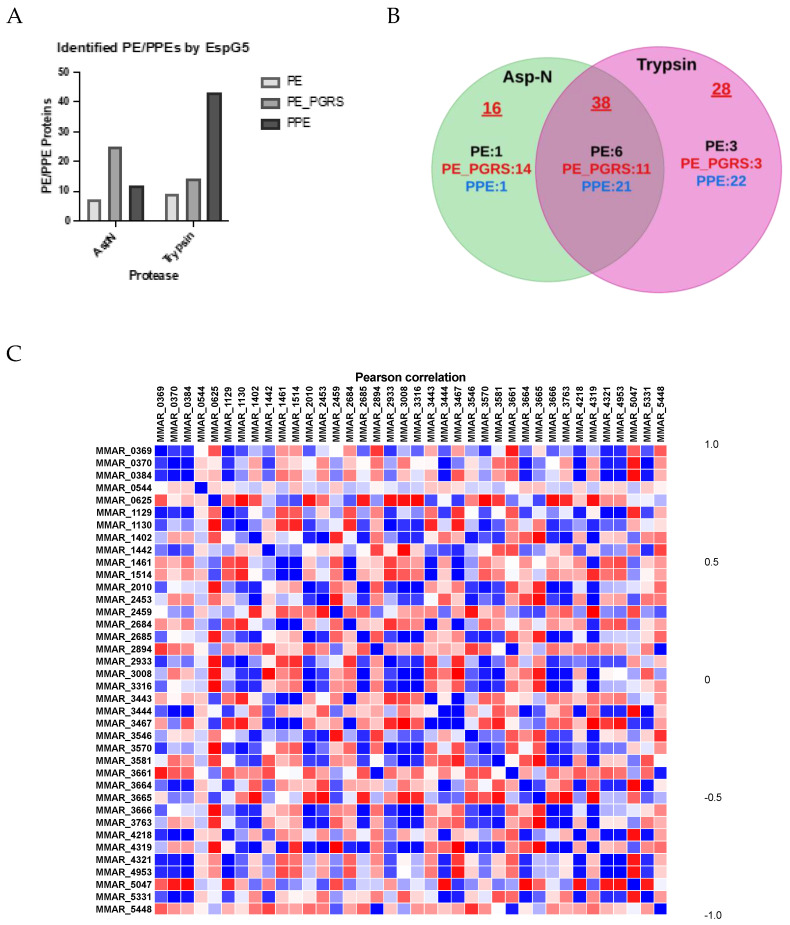
Isolation of PE/PPE proteins from *M. marinum* culture filtrate by EspG5 pulldown. (**A**) Identification of EspG5—interacting PE/PPE proteins by AspN— and trypsin—digested profiles is plotted. (**B**) Venn diagram demonstrates that 38 EspG5—interacting PE/PPE proteins can be found from AspN— [31] or trypsin (purple) —treated samples. The numbers of PE/PPE proteins that were uniquely found in AspN and trypsin digests are indicated. (**C**) Pearson correlation of PE—PPE protein interactions. The correlation of the interaction is presented by the color spectrometry from red (low) to blue (high).

We further characterized the substrate spectrum of EspG5 by pulldown assay. We immobilized GST-EspG5 as a bait to isolate its interacting partners from the culture filtrate of *M. marinum*. EspG5, and its bound proteins were released from the GST tag by PreScission protease cleavage. A total of 82 PE/PPE proteins (10 PE, 28 PE_PGRS, and 44 PPE) were mapped (Figure 3A,B). Of them, 7 and 9 PE, 25 and 14 PE_PGRS, and 22 and 43 PPE were shown in the AspN- and trypsin-digested profiles, respectively. While both protease treatments gave a similar number of PE identification, the number of PE_PGRS found in AspN profile was almost twice than that in the trypsin profile. On the contrary, trypsin digestion favored PPE identification. The PPE proteins bound to EspG5 accounted for approximately 85% of the secreted PPE proteins. We observed that PPE15 bound to EspG5 (Table S5), as reported previously [21]. With reference from the Mycobrowser database [11,13,19], 55 PE/PPE identified in the EspG5 interactome here were ESX-5 associated. Interestingly, MMAR_5448 and PE5 (MMAR_0544), from the ESX-1 and ESX-3 secretion systems, respectively, were also detected in the EspG5 pulldown.

### 2.4. Mapping PE/PPE Protein Networks

From the pulldown assay, we found that EspG5 substrates included several PE/PE_PGRS, suggesting that PE proteins coeluted with cognate PPE partners. Given that the abundance of PE/PPE pairs was similar, we performed Pearson correlation analysis to deduce their relationship. Protein abundance data from AspN and trypsin treatments were combined to minimize the influence of detectable peptides generated by enzymatic cleavage and, thus, improve the accuracy of the correlation analysis (Figure 3C). Forty PE/PPE pairs and fifty PE_PGRS/PPE pairs with Pearson correlation coefficients higher than 0.9 were selected. These pairs involved 29 PPE, 9 PE, and 17 PE_PGRS proteins, suggesting that PPE proteins have multiple PE and PE_PGRS partners. To enhance the confidence level of the analysis, we increased the threshold of the correlation coefficient to 0.95 and obtained 36 PE/PPE and 24 PE_PGRS/PPE pairs. Additional selection criteria were adopted, including the predictive paralog matching score (Sij) and the coexpression score (Rij) [39], according to the Gene Expression Omnibus database [38,40,41]. The analysis identified 12 pairs with Sij greater than 0.75 and Rij greater than 0.35 (Table 2). Among them, the MMAR_5448-MMAR_2894 pair has been characterized previously [14]. Other binding partners included PPE40 (MMAR_3666) with PE_PGRS (MMAR_1208, MMAR_3316, and MMAR_2010), PPE30 (MMAR_2685) with PE_PGRS (MMAR_1208 and 2010), and PPE61 (MMAR_3444) with PE (MMAR_0370 and 3467). The presence of more than one PE/PE_PGRS partner was supported by the high sequence homology of the N-terminal domains of PEs; for instance, there was 99% in MMAR_1208 and MMAR_3316, 51% in MMAR_1208 and MMAR_2010, and 45% in MMAR_0370 and MMAR_3467.

### 2.5. Interaction of EspG5 with PPE31 and PPE32

**Figure 4:**
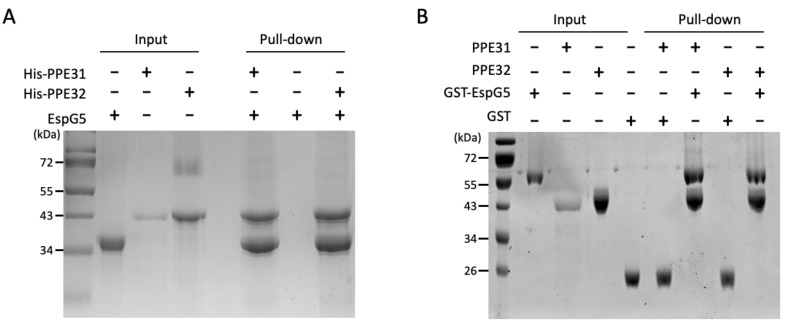
Molecular interaction of GST—EspG5 with His—PPE31 and —PPE32 by pulldown assay. Purified proteins were loaded to (**A**) nickel affinity beads (**B**) glutathione sepharose beads. The bound proteins were analyzed using SDS—PAGE and Coomassie stained. In (**B**), GST was used as a control.

**Figure 5:**
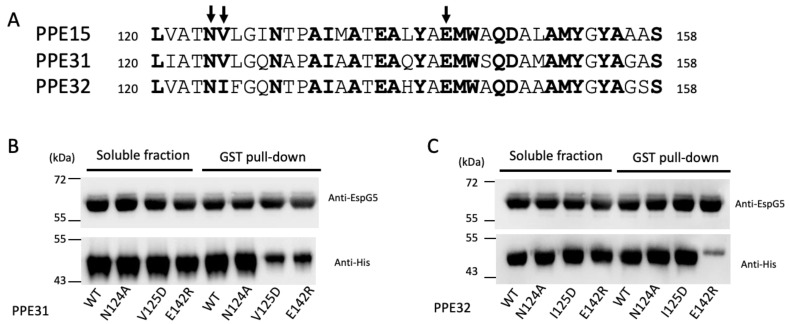
Mutagenesis study. (**A**) Sequence alignment between PPE15, PPE31 and PPE32. The sequence of the binding interface of EspG5 and PPE15 was analyzed. Mutation sites in this study were indicated by arrow. (**B**,**C**) Effect of these mutations on the EspG5 interactions were studied using pulldown assay and western analysis.

Next, we validated the binding of EspG5 with PPE31 and PPE32. These two PPE proteins have been proposed to be ESX-5 associated; however, their biophysical interactions with EspG5 have never been reported [26,42]. We purified His-tagged PPE31 and PPE32, and EspG5 with or without GST tag for nickel and GST pulldown assays. As shown in Figure 4, EspG5 interacted directly with PPE31 and PPE32. We questioned that their binding mode may share some similarities with the structurally solved EspG5-PPE15 pair [21]. From the multiple sequence alignment (Figure 5A), equivalently conserved residues Asn124, Val125/Ile125, and Glu142 on the EspG5-binding interface were selected for mutagenesis study. The solubility of these His-tagged PPE mutants, however, was very low when expressed individually in *E. coli*. Instead, their solubility was improved upon coexpression with GST-EspG5. GST pulldown assays were carried out using the coexpressed clear lysate. The results indicated that mutations V125D and E142R in PPE31 reduced the interaction with EspG5 (Figure 5B,C). Interestingly, for PPE32, only the E142R mutant exhibited an inhibitory effect towards EspG5 binding, suggesting that the conserved binding mode of EspG5 and PPE lies on residue Glu 142.

**Figure 6:**
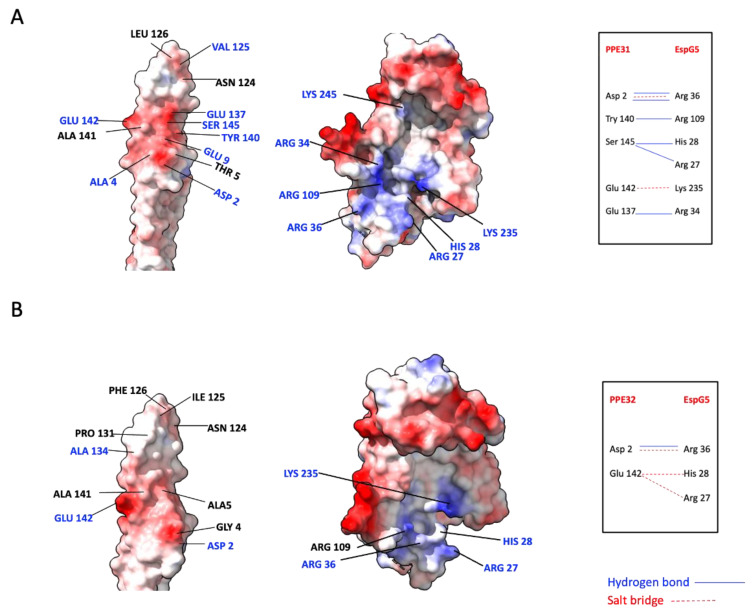
Homology models of EspG5 in complex with PPE31 (**A**) and PPE32 (**B**). (**Left**) The ‘open book’ views of the structural interface. The PPE proteins and EspG5 molecular surfaces are colored by electrostatic potential. The residues on the binding interface are labelled. (**Right**) Residues forming hydrogen bonds (––) and salt bridges (---) on the interface are indicated.

We attempted to crystallize these EspG5/PPE complexes but without success. Therefore, the N-terminal PPE domain of PPE31 and PPE32 were predicted using AlphaFold 2 [43]. This was followed by molecular docking using the known structure of EspG5 (PDB: 5XFS) by ClusPro 2 [44]. Similar to PPE15, residues Asn124 and Val125/Ile125 are at the tip of the PPE domain while Glu142 is at the central core of the structure. Analysis of the EspG5-PPE31 and EspG5-PPE32 homology models suggested that their binding interfaces were dominated by hydrophobic and electrostatic interactions (Figure 6). In particular, patches containing negatively charged residues (Asp2, Glu137, and Glu142) on PPE helped stabilize the heterodimer, consistent with the mutagenesis and pulldown results showing that Glu142 was critical in EspG5 interaction. However, it appears that the number of hydrogen bonds and salt bridges involved in EspG5-PPE32 interaction is less than that in EspG5-PPE31 (Figure 6A,B).

## 3. Discussion

PE/PPE proteins are substrates of the ESX secretion system of mycobacteria, and some of them function in pairs. Despite the highly variable C-terminal region, the conserved N-terminal domain and the limited number of arginine and lysine residues hinder their identification using trypsin-based proteomic approaches. This study analyzed the proteome of *M. marinum* using trypsin and AspN. Collectively, our results identified 136 PE/PPEs (13 PE, 71 PE_PGRS, and 52 PPE proteins), representing 48% of the PE/PPE proteome. Forty PE/PPE proteins have been reported in previous studies [4,26]; these include

PE5, involved in iron acquisition [3]; the PE_PGRS MMAR_3581, involved in actin-based motility by recruiting the host nucleation-promoting factor N-WASP [42]; and PPE64, involved in heme binding [45]. Notably, 27 PE/PPE proteins are specifically shown in the AspN profile, and many of them have not been mapped before [4,26]. These include PE_PGRS2 (MMAR_4561) and the PE MMAR_0111, which contain one trypsin cleavage site each and 22 and 7 AspN cleavage sites, respectively. These results suggest the importance of using AspN to obtain a comprehensive PE/PPE profile, particularly for PE_PGRS proteins, which are dominant in the PE family and are highly polymorphic. On the other hand, we analyzed the 22 PPE proteins uniquely found from trypsin-treated profile (Figure 3A). We found that the number of tryptic cleavage sites is higher than that of the AspN sites in some of these PPE proteins (e.g., MMAR_3578, MMAR4202). For some other PPE such as MMAR_0685 and MMAR_1950, although the numbers of tryptic and AspN cleavage sites are similar, the distribution of these tryptic sites along the sequence allows the generation of peptide fragments with better *m*/*z* range for detection. Taken together, when combining with the trypsin digestion, a more precise quantitation of the PE/PPE expression level can be achieved.

Transcriptional expression of *pe/ppe* genes is under tight control and relates to the culture conditions and environmental stresses. Previous studies have shown that MMAR_1161 and MMAR_3465 are weakly expressed, but they are upregulated in a mutant strain defective in transcription factor espM and under hypoxic conditions, respectively [41]. However, detecting small PE/PPE proteins that only contain the N-terminal PE or PPE domains is challenging. For instance, PE19 (MMAR_2673; 100 residues), which has one AspN cleavage site and no trypsin cleavage sites, is highly expressed [38] but was not found in our study.

The present study also reports a PE/PPE-EspG5 network consisting of 10 PE, 28 PE_PGRS, and 44 PPE proteins. ESX-5 is responsible for the export of most PE/PPE proteins, and it has been predicted that 95% of PE/PPE proteins have the potential to interact with EspG5 [17]. Some of these predicted PPE proteins including PPE15, PPE25, PPE26, PPE31, and PPE32 are also identified in our in vitro pulldown experiments. *ppe25* and *ppe26* genes are in the *esx-5* locus [46]. We confirm EspG5 interacts directly with PPE31 and PPE32. The formation of these binary complexes resembles that of EspG5/PPE15, in which the conserved residue Glu142 on PPE are critical for binding. We examined the EspG5-PPE31/PPE32 homology models. Both Val 125 in PPE31 and Ile125 in PPE32 are in a flexible loop of the tip of the PPE domain. In PPE31, this loop is entirely embedded in the hydrophobic pocket of EspG5 so that interactions between Val125_PPE31_ and Phe191_EspG5_ and Leu216_EspG5_ were found. Interestingly, in the PPE31-EspG5 model, PPE31 was docked in a tilted orientation. Although the central interface via Glu142 in PPE31 was retained, but Ile125 was not involved in any hydrophobic interaction with EspG5. We noted that, in the PPE32 sequence, next to residue Ile125 is a phenylalanine. It is possible that insertion of the flexible loop containing a bulky side chain of Phe126 into the EspG5 hydrophobic pocket would shift the whole PPE domain along the binding interface and, subsequently, prevent the formation of charge–charge interactions of Glu142_PPE32_ with His28_EspG5_ and Arg287_EspG5_, which is more critical in EspG5–PPE interaction. These may explain the different effects of Val125 in PPE31 and Ile125 in PPE32 in EspG5 binding. The interactions between EspG5 with PPE31 and PPE32 are also supported by a study using a *ppe38* mutant strain that inhibited the secretion of two large subsets of ESX-5 substrates, PPE/MPTR and PE_PGRS [26]. Moreover, PPE31 modulates innate immune responses [47], indicating that this protein is secreted. Interestingly, EspG cross-reacts with PPE proteins from another ESX cluster, because ESX clusters are conserved and encode paralogous type VII secretion systems [17,31]. Three of the eighty-two PE/PPE proteins isolated from EspG5 pulldown assays were associated with ESX-1 and ESX-3. For instance, the MMAR_2894/PPE68 pair, located in the *esx-1* locus, was highly abundant in the EspG5 interaction network.

Correlation analysis based on protein abundance, phylogenetic relationships, and coexpression generates a list of potential PE/PE_PGRS/PPE pairs. Apart from the well-characterized MMAR_2894/PPE68 pair [4,14], some PPEs interact with multiple PE/PE_PGRS so that their PE domains share high sequence similarity. It is noteworthy that the number of *pe/pe_pgrs* genes is larger than the number of *ppe* genes in the genome, and recombinant PE/PPE proteins are often unstable when expressed alone. These suggest that one PPE may have multiple PE/PE_PGRS partners and give rise to different biological functions. Given the high similarity of the *M. marinum* and *Mtb* genomes, the application of this modified proteomic approach to determine the expression and localization of PE/PPE proteins under different growth conditions and infection stages will improve our understanding of this protein family.

## 4. Materials and Methods

### 4.1. Bacterial Strain and Growth Condition

The *Mycobacterium marinum M* strain cells were inoculated onto Middlebrook 7H10 agar (Becton, Dickinson, NY, USA) plates at 32 °C in the dark in a biological safety level 2 laboratory (BSL2). After 5 days, colonies were harvested and inoculated into a 5 mL Middlebrook 7H9 culture medium supplemented with 10% Middlebrook OADC (Becton, Dickinson, NY, USA) and 0.05% Tween 80 as a starter. When the OD600 of the starter reached 0.6 to 0.8, it was inoculated into 400 mL Sauton’s medium supplemented with 0.05% Tween 80. Cells were harvested for further analysis when the OD600 was 0.8 to 1.0. Briefly, mycobacterial cells were centrifuged at 2700× *g* for 10 min at 4 °C. The supernatant was filtered using 0.22 μm filter units.

### 4.2. Preparation of Culture Filtrate

The *M. marinum* culture filtrate was concentrated using 3K PIERCE™ PROTEIN concentrator (Themo Fisher Scientific, Waltham, MA, USA). The 200 mL filtered culture filtrate was concentrated to 5 mL followed by precipitation by prechilled acetone at −30 °C overnight. The acetone precipitated culture filtrate was centrifuged at 15,000 rpm for 15 min at 4 °C, and the pellet was washed with 2 mL of prechilled acetone before LC-MS analysis.

### 4.3. Cloning and Purification of the Recombinant Proteins

The PPE31 and PPE32 genes were amplified from H37Rv genomic DNA and cloned into a pAC28m vector with a C-terminal His tag. For the construction of PPE31 and PPE32 mutants, the QuickChange site-directed mutagenesis kit (Stratagene Corp., La Jolla, CA, USA) was employed according to the manufactural protocol. After confirmation of the DNA sequences, the recombinant PPE proteins were expressed in *E. coli* BL21(DE3). The protein expression was induced with 0.5 mM isopropyl-β-d-thiogalactoside (IPTG) at OD600 of 0.6–0.8, and the culture was maintained at 16 °C overnight. The cell pellets were resuspended in buffer A (20 mM HEPES pH 7.5, 500 mM NaCl, and 0.2 mM PMSF and 0.2 mM benzamidine) containing 2% N-lauroylsarcosine sodium salt to solubilize the PPE proteins. After sonication, the clear lysate was loaded to nickel sepharose, and then the column was washed with 10 column volumes (CV) of lysis buffer. The PPE proteins were eluted in buffer B (20 mM HEPES pH 7.5, 500 mM NaCl, 200 mM imidazole, 0.2 mM PMSF, and 0.2 mM benzamidine). The protein samples were further purified with Superdex 75 (GE Healthcare) size-exclusion columns pre-equilibrated with buffer C (20 mM HEPES pH 7.5, 150 mM NaCl, 0.2 mM PMSF, and 0.2 mM benzamidine). The expression and purification of EspG5 was carried out as described in [21].

### 4.4. Pulldown Assays

For the identification of EspG5 interacting partners, 200 mL of *M. marinum* culture filtrate was concentrated to 2 mL in the buffer containing 250 mM NaCl, 20 mM HEPES, 2 mM DTT, 0.2 mM benzamidine, and 0.2 mM PMSF for the pulldown assay. Forty ug purified GST and GST-EspG5 were immobilized with 40 µL glutathione-Sepharose 4B beads (50% slurry) for 2 h at 4 °C with rotation. The beads were washed five times, followed by incubation with concentrated culture filtrate for another 2 h. After washing, PreScission protease was added to release the bound EspG5 and its interacting partners from the GST-tag. The supernatant was collected for further mass spectrometry.

To validate the interaction of EspG5 with PPE31 and PPE32, a pulldown assay using purified GST-EspG5, His-PPE31, and His-PPE32 was conducted in a buffer containing 20 mM HEPES, pH 7.5, 250 mM NaCl, 0.2 mM PMSF, and 0.2 mM benzamidine. In the GST pulldown assay, purified GST-EspG5 or GST protein was immobilized onto glutathione beads. After washing, purified PPE31 and PPE32 were added, and the mixture was incubated for 2 h. On the other hand, purified His-PPE31 and His-PPE32 were immobilized onto nickel beads and further incubated with EspG5. After washing, the bound proteins were analyzed using SDS-PAGE and Coomassie stained.

PPE31 and PPE32 mutants were insoluble when expressed individually. They were therefore coexpressed with GST-tagged EspG5. The clear lysate in the buffer containing 20 mM HEPES, pH 7.5, 250 mM NaCl, 0.2 mM PMSF, and 0.2 mM benzamidine was mixed with glutathione agarose and incubated for 2 h at 4 °C. After washing, the immobilized beads were analyzed using SDS-PAGE followed by western analysis using anti-His and anti-EspG5 antibodies. The latter was generated using an in-house antibody production service.

### 4.5. Protease Digestion

Protein samples were denatured by adding urea to a final concentration of 8 M, followed by cysteine reduction by adding DTT to a final concentration of 0.5 M and incubation at 56 °C for 45 min; 0.6 M iodoacetamide was added and incubated for 30 min at room temperature in the dark for the alkylation. Before protease digestion, the urea was diluted to 2 M with 50 mM of ammonium bicarbonate buffer for trypsin digestion or 50 mM tris buffer for AspN digestion, respectively. Then, the samples were digested in 37 °C for 16 h using trypsin or AspN (Promega, Madison, WI, USA), respectively. Samples were desalted using Pierce™ C18 Spin Tips (Thermo Scientific, Waltham, MA, USA), according to the manufacturer instructions, and reconstituted in 0.1% formic acid (*v*/*v*) in milliQ-H_2_O for injection.

### 4.6. Mass Spectrometry

The peptide samples were analyzed using the Orbitrap Fusion Lumos Tribrid Mass Spectrometer (Themo Fisher Scientific, Waltham, MA, USA). To separate 200 ng of peptides, the injections were performed at 1 mL/min flow rate in 98% Milli-Q/2% ACN + 0.1% formic acid (A) and 98% Acetonitrile ACN/2% Milli-Q + 0.1% formic acid (B) buffer for 10 min. The mass spectrometry was acquired in the Orbitrap at a resolution 60,000 resolution with the mass range of 375 to 1500 *m*/*z*. Fragmentation was selected using the Monoisotopic Peak Determination (MIPS) and precursors with charge states between 2 and 7 with the reporter ion intensity threshold setting higher than 5000. In-source CID (collision-induced dissociation) was set to the fixed mode, with 30% collision energy and 10 ms activation time. The MS spectra were acquired under the Orbitrap full scan resolution of 30,000 and a maximum injection time of 100 ms. The error tolerances were set at 10 ppm for the precursor mass, and dynamic exclusion was set to 60 s after 1 count.

### 4.7. LC-MS Data Analysis

Proteome Discoverer 2.4.0.305 software (Thermo Fisher Scientific, Waltham, MA, USA) was used to search RAW files against *Mycobacterium marinum M* databases (MycoBrowser portal, released in June 2018). The MS precursor filter was set at 350Da to 5000Da. Peak lists were generated with the spectrum selector node (default parameters) of Proteome Discoverer. And MS spectra to database matching was carried out using the SEQUEST-HT algorithm. Peak filters were set as S/N threshold (FT-only), 1.5. Either full trypsin or AspN was chosen appropriately. The proteolytic enzyme specificity with two possible missed cleavage sites, methionine oxidation/+15.995 Da (M), and N-acetylation/+42.011 Da (N-terminus) was set as variable modifications. Cysteine carbamidomethylating/+57.021 Da (C) was set as fixed modifications. In spectrum matching, neutral loss of a ions, b ions, y ions and flanking ions were used with weight of a ions: 0, weight of b ions: 1, weight of c ions: 0, weight of x ions: 0, weight of y ions: 1, and weight of z ions: 0. The tolerance for the precursor mass and fragment mass searches was 10 ppm and 0.02 Da, respectively. All MS spectra were searched using SEQUEST combined with the Percolator algorithm with a maximum Delta Cn parameter 0.05 for PSM filtering. Only high confidence peptides of the target false discovery rate (FDR) between 0.01(strict) and 0.05 (relaxed) with validation based on the q-Value were finally considered.

Label-free quantifications (LFQ) were applied for the PE/PPE protein abundance analysis. Protein abundance was calculated by the sum of all unique peptide ion abundances for a specific protein in each run. Normalization and scale were executed by Log2(Abundance) of their source proteins, as detected in proteomic analysis. The phylogenetic analysis was performed with CLUSTALW. Branches showed the paths by which genetic information was passed from one generation to the next. Typically, the extent of genetic change was measured by estimating the average number of protein substitutions per site.

### 4.8. Western Analysis

Coexpressed GST-EspG5 with His-PPE31 and His-PPE32 samples were subjected to GST pulldown. The results were analyzed using SDS-PAGE and western analysis. Anti-6XHis (Abcam, Cambridge, UK; 1:5000) and anti-EspG5 (produced in-house) antibodies were used for detection of His-tagged PPE proteins and GST-EspG5, respectively. Donkey anti-rabbit and goat anti-mouse IgM secondary antibodies (Santa Cruz, CA, USA; 1:2000) were used.

### 4.9. Structural Model Prediction

Structures of the PPE domain of PPE31 and PPE32 were predicted with AlphaFold 2 [43]. In brief, the N-terminal 180 amino acid sequence was uploaded to the AlphaFold Colab server. MSA-method mmseqs2 and other default parameters were used. Models of PPE31 and PPE32 with highest pLDDT score were used for docking. ClusPro 2 was employed for the docking between PPE proteins and EspG5 based on the PIPER algorithm [44]. The structure of EspG5 was obtained from Protein Data Bank (PDB: 5XFS). PPE31 and PPE32 were set as potential ligands, whereas EspG5 was set up as a receptor. The binding interface of the predicted complexes were analyzed using PDBsum. ChimeraX was used for model alignment (https://www.rbvi.ucsf.edu/chimerax; accessed on 22 November 2022).

### 4.10. Phylogenetic Tree and PE–PPE Pairs Calculation

A phylogenetic tree was generated from the PE/PPE subalignment using ClusterW [48]. PE–PPE pairs were calculated with the previous method [38]. Briefly, distance matrices X and Y were constructed for PE and PPE of the *M. marinum*, respectively. These matrices represented the percent divergence between pairs of proteins within each family. Secondly, the correlation between the ordered distance matrices X and Y was calculated using the Pearson correlation coefficient. This correlation quantified the coevolution between PE and PPE proteins. Thirdly, different prediction thresholds ranging from 0.0 to 1.0 were evaluated. Finally, PE/PPE pairs with a correlation score (Sij) above the prediction threshold were selected as potential interacting partners. A threshold of approximately 0.75 was chosen. Gene expression datasets for *M. marinum* were collected and concatenated to create expression vectors for each gene [25,41]. Correlation coefficients of gene expression vectors are calculated to measure the coexpression of genes.

### 4.11. Statistical Analysis

All statistical analyses were processed as described in figure legend by GraphPad prism 9 (California, USA). The differences between two groups were analyzed using the Student’s t test. The *p* value < 0.05 was considered a statistical significance.

## Figures and Tables

**Table 1:** The number of the PE, PE_PGRS, and PPE proteins identified by AspN and/or trypsin treatment.

	PE	PE_PGRS	PPE	Total
AspN unique	3	18	6	27
Trypsin unique	1	14	8	23
AspN/Trypsin	9	39	38	86
Total (% PE/PPE)	13 (48.1%)	71 (47.8%)	52 (49.1%)	136 (48.4%)

“AspN unique” and “Trypsin unique” indicate the number of PE/PPE proteins that were only found in AspN- and trypsin-treated samples, respectively. The “AspN/Trypsin” indicates the number of PE/PPE proteins that were found in both AspN-treated samples and trypsin-treated samples.

**Table 2:** List of the top 12 possible PE-PPE pairs. These pairs have been selected based on their coefficients, where a threshold of 0.95 was applied. Additionally, the pairs needed to exhibit S_ij_ values above 0.75 and R_ij_ values over 0.35.

PE_ORF1	PE_GENE1	PPE_ORF2	PPE_GENE2	Coefficient	S_ij_	R_ij_
MMAR_3763	PE_PGRS27	MMAR_1950	PPE15_1	0.99849	0.770717	0.673922
MMAR_1208	PE_PGRS	MMAR_3666	PPE40	0.998099	0.894426	0.588773
MMAR_3467	PE	MMAR_1514	PPE51_1	0.997299	0.775825	0.65997
MMAR_3316	PE_PGRS	MMAR_3666	PPE40	0.996912	0.887535	0.604463
MMAR_2010	PE_PGRS	MMAR_2685	PPE30	0.996684	0.797027	0.478634
MMAR_3467	PE	MMAR_3444	PPE61	0.996225	0.787156	0.495639
MMAR_2010	PE_PGRS	MMAR_3666	PPE40	0.988286	0.795013	0.582921
MMAR_2894	PE	MMAR_5448	PPE68	0.982382	0.974785	0.784697
MMAR_1208	PE_PGRS	MMAR_2685	PPE30	0.980908	0.89644	0.480968
MMAR_0370	PE	MMAR_4218	PPE	0.976292	0.81602	0.605133
MMAR_3316	PE_PGRS	MMAR_4319	PPE13	0.966084	0.891999	0.35109
MMAR_0370	PE	MMAR_3444	PPE61	0.964489	0.906339	0.499527

## Data Availability

All the LC-MS raw data were submitted to the PRIDE databank, with the PXD050612 identifier.

## Supplementary Materials

The following supporting information can be downloaded at: https://www.mdpi.com/article/10.3390/ijms25179550/s1.
