# Supplementary material for: PE/PPE Proteome and ESX-5 Substrate Spectrum in Mycobacterium marinum
Source: Int J Mol Sci. 2024 Sep 3;25(17):9550. doi: 10.3390/ijms25179550 (PMC11395111; doi:10.3390/ijms25179550)
Supplement: Supplementary file 1 [file ijms-25-09550-s001.zip › Figure S1.pptx]

## Slide 1
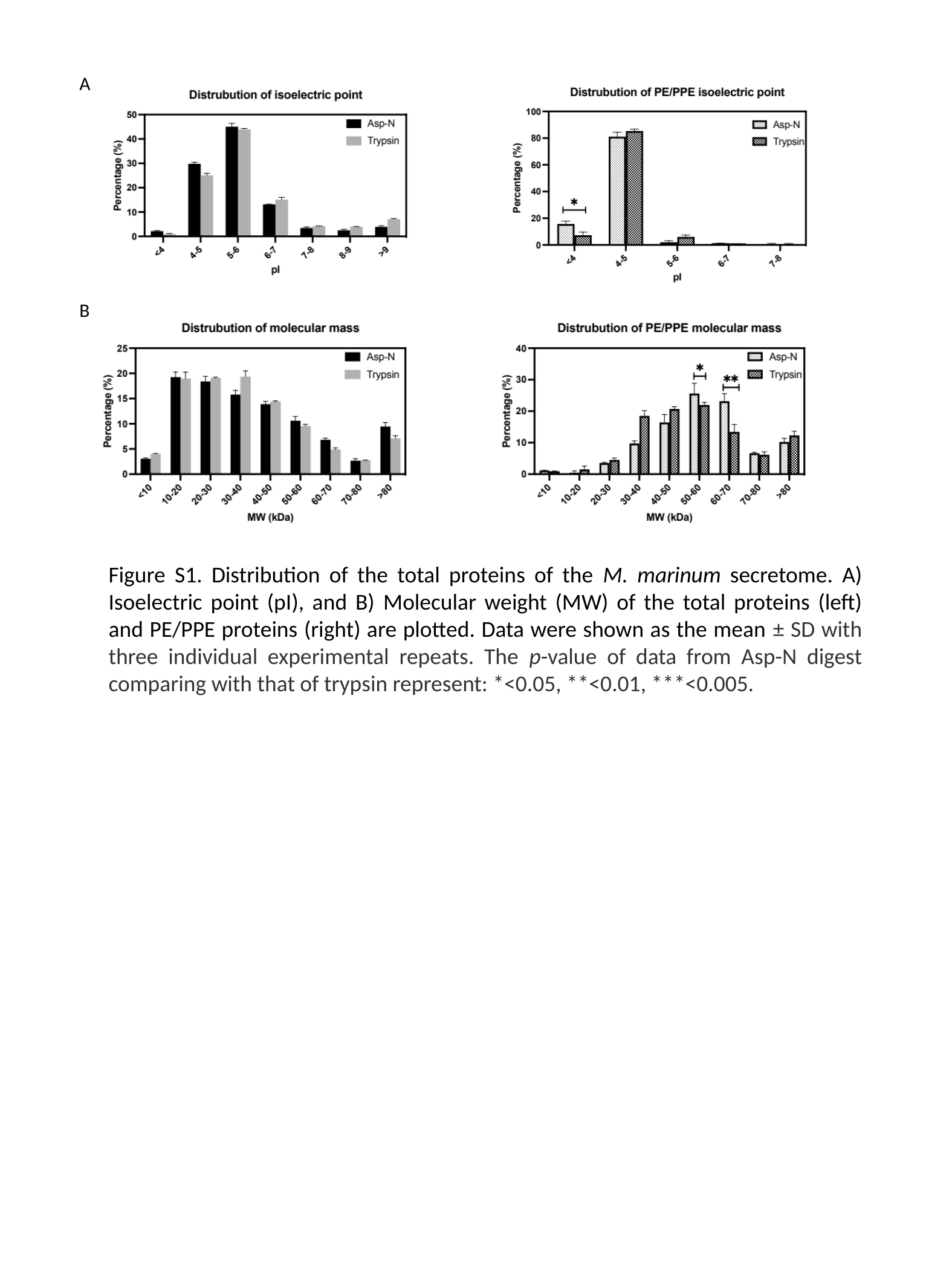

A
B
Figure S1. Distribution of the total proteins of the M. marinum secretome. A) Isoelectric point (pI), and B) Molecular weight (MW) of the total proteins (left) and PE/PPE proteins (right) are plotted. Data were shown as the mean ± SD with three individual experimental repeats. The p-value of data from Asp-N digest comparing with that of trypsin represent: *<0.05, **<0.01, ***<0.005.
