# Supplementary figures and images for: PE/PPE Proteome and ESX-5 Substrate Spectrum in Mycobacterium marinum
Source: Int J Mol Sci. 2024 Sep 3;25(17):9550. doi: 10.3390/ijms25179550 (PMC11395111; doi:10.3390/ijms25179550)

Full original images of western blots indicated in the figure 5 B (top) & C (bottom).

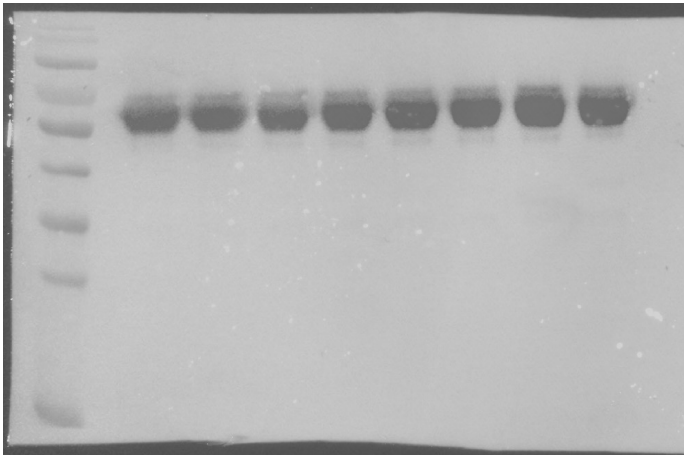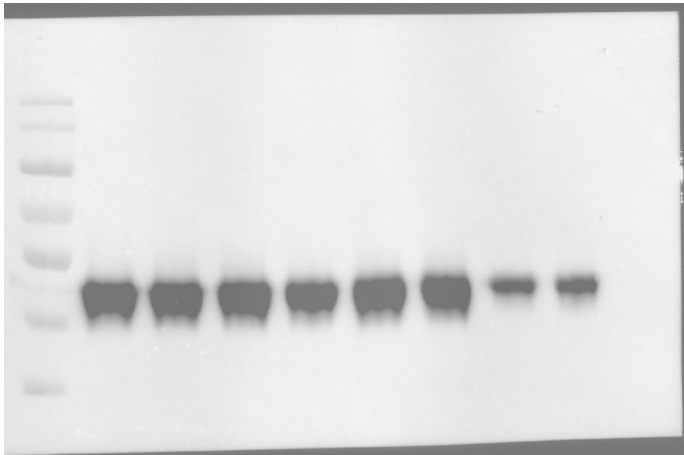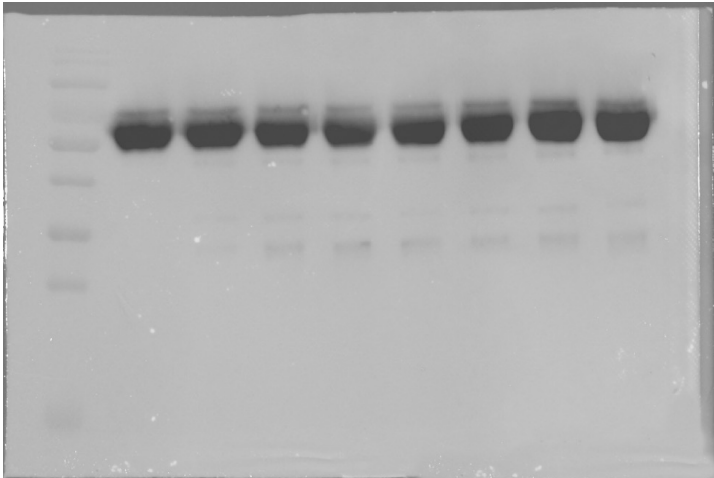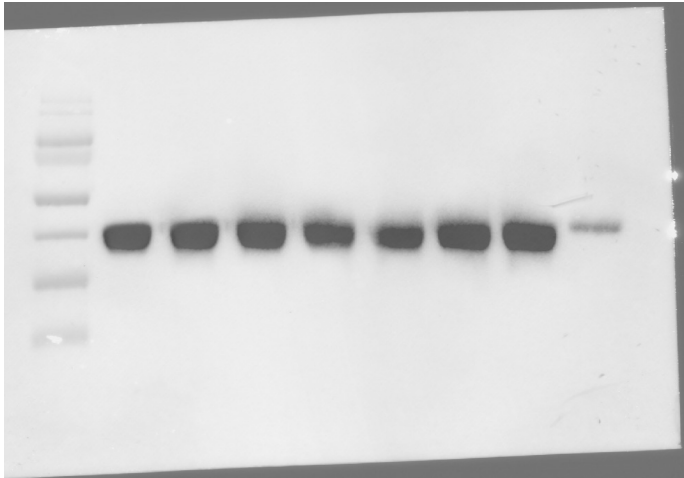

Supplement: Supplementary file 1 [file ijms-25-09550-s001.zip › original_blot.pdf]
